# Risk factors for one-year mortality in 440 femoral peri-implant fractures: insights from the PIPPAS prospective, multicentre, observational study

**DOI:** 10.1302/2633-1462.61.BJO-2024-0113.R1

**Published:** 2025-01-09

**Authors:** Héctor J. Aguado, The PIPPAS Study Group

**Affiliations:** 1 Hospital Clínico Universitario de Valladolid, Valladolid, Spain; 1 Hospital Clínico Universitario de Valladolid, Valladolid, Spain; 2 Hospital Universitari Mútua de Terrassa, Barcelona, Spain; 3 Hospital Universitari Vall d´Hebrón de Barcelona, Barcelona, Spain; 4 Hospital Fundació Althaia de Manresa, Barcelona, Spain; 5 Hospital Clínic de Barcelona, Barcelona, Spain; 6 Hospital 12 de Octubre de Madrid, Madrid, Spain; 7 Hospital de Jove de Gijón (Asturias), Oviedo, Spain; 8 Hospital Universitario de Basurto, Bizkaia, Basurto, Spain; 9 Hospital Álvaro Cunqueiro de Vigo, Pontevedra, Spain; 10 Hospital Universitario de Galdakao-Usansolo, Bizkaia, Galdakao-Usansolo, Spain; 11 Hospital Universitario de Canarias de Tenerife, Tenerife, Spain; 12 Hospital Universitario Dr. Peset de Valencia, Valencia, Spain; 13 Hospital Royo Villanova de Zaragoza, Zaragoza, Spain; 14 Hospital Universitario de Cabueñes de Gijón, Gijón, Spain; 15 Hospital Clínico San Carlos de Madrid, Madrid, Spain; 16 Complejo Hospitalario Universitario de A Coruña, Coruña, Spain; 17 Hospital General Universitario Gregorio Marañón de Madrid, Madrid, Spain; 18 Hospital Universitario de Álava, Vitoria-Gasteiz, Spain; 19 Hospital Universitario General de Elche, Elche, Spain; 20 Hospital General Universitario Los Arcos del Mar Menor de Murcia, Murcia, Spain; 21 Hospital Vega Baja de Orihuela, Alicante, Alicante, Spain; 22 Hospital General Universitario J.M. Morales Meseguer de Murcia, Murcia, Spain; 23 Hospital Universitario Marqués de Valdecilla de Santander, Santander, Spain; 24 Hospital Universitario de Toledo, Toledo, Spain; 25 ospital Puerta de Hierro de Majadahonda, Madrid, Madrid, Spain; 26 Complejo Asistencial de Segovia, Segovia, Spain; 27 Consorci Sanitari Integral - Hospital Sant Joan Despí- Moisès Broggi de Barcelona, Barcelona, Spain; 28 Hospital Universitari Sagrat Cor - Quirónsalud de Barcelona, Barcelona, Spain; 29 Complejo Hospitalario de Llerena-Zafra, Badajoz, Spain; 30 Hospital Universitario Lucus Augusti de Lugo, Lugo, Spain; 31 Hospital Ramón y Cajal de Madrid, Madrid, Spain; 32 Hospital Príncipe de Asturias de Alcalá de Henares, Madrid, Spain; 33 Hospital General Universitario Reina Sofía de Murcia, Murcia, Spain; 34 Hospital Universitario de Guadalajara, Guadalajara, Mexico; 35 Hospital Universitari Doctor Josep Trueta de Girona, Girona, Spain; 36 Complejo Hospitalario Universitario de Albacete, Albacete, Spain; 37 Hospital Sierrallana de Torrelavega, Cantabria, Torrelavega, Spain; 38 Complejo Asistencial Universitario de Palencia, Palencia, Spain; 39 Hospital Universitario Fundación Jiménez Díaz de Madrid, Madrid, Spain; 40 Complexo Hospitalario Universitario de Pontevedra, Pontevedra, Spain; 41 Hospital Universitari Parc Taulí de Sabadell, Barcelona, Barcelona, Spain; 42 Hospital Universitario Miguel Servet de Zaragoza, Zaragoza, Spain; 43 Hospital Universitario Arnau de Vilanova de Lleida, Lleida, Spain; 44 Complejo Asistencial Universitario de Burgos, Burgos, Spain; 45 Hospital Universitario San Pedro de Logroño, Logroño, Spain; 46 Hospital Universitario Costa del Sol de Marbella, Málaga, Marbella, Spain; 47 Hospital Parc De Salut Mar de Barcelona, Barcelona, Spain; 48 Hospital Italiano de Buenos Aires, Argentina, Argentina, Spain; 49 Hospital Universitario de Salamanca, Salamanca, Spain; 50 Hospital Universitari Sant Joan de Reus, Tarragona, Spain; 51 Hospital Virgen del Rocío de Sevilla, Sevilla, Spain; 52 Hospital de la Santa Creu i Sant Pau de Barcelona, Barcelona, Spain; 53 Hospital Universitario Infanta Leonor de Madrid, Madrid, Spain; 54 Hospital Universitario de Donostia, Donostia, Spain; 55 Hospital Reina Sofía de Tudela, Navarra, Tudela, Spain; 56 Hospital San Agustín de Avilés, Asturias, Spain; 57 Hospital Universitario Virgen Macarena de Sevilla, Sevilla, Spain

**Keywords:** Peri-implant fracture, Femur, Outcome, Mortality, Replacement, Fracture fixation, geriatric co-management, Incidence, epidemiology, management, frailty, peri-implant fractures, Charlson Comorbidity Index (CCI), surgical approaches, arthroplasties, univariate analyses, Anesthesiologists, cognitive impairment, anticoagulant, multivariate analyses, Medical complications

## Abstract

**Aims:**

The Peri-Implant and PeriProsthetic Survival AnalysiS (PIPPAS) study aimed to investigate the risk factors for one-year mortality of femoral peri-implant fractures (FPIFs).

**Methods:**

This prospective, multicentre, observational study involved 440 FPIF patients with a minimum one-year follow-up. Data on demographics, clinical features, fracture characteristics, management, and mortality rates were collected and analyzed using both univariate and multivariate analyses. FPIF patients were elderly (median age 87 years (IQR 81 to 92)), mostly female (82.5%, n = 363), and frail: median clinical frailty scale 6 (IQR 4 to 7), median Pfeiffer 4 (1 to 7), median age-adjusted Charlson Comorbidity Index (CCI) 6 (IQR 5 to 7), and 58.9% (n = 250) were American Society of Anesthesiologists grade III.

**Results:**

Overall, 90.5% (n = 398) of the patients were treated surgically, 57.0% (n = 227) retained the implant, and 88.7% (n = 353) managed with fixation. Mortality rates were 8.2% (n = 3.6) in-hospital, 11.4% (n = 50) at 30 days, 21.1% (n = 93) at six months, and 21.6% (n = 95) at 12 months. Medical complications, mainly delirium, were common in the acute setting (52.7%, n = 215). The nonunion rate was 4.1% (n = 18). Mortality risk factors in the univariate analysis were age, living at a nursing home, no walking outdoors, frailty variables, fractures in the distal epiphysis, fractures around a proximal nail, discharge to a healthcare facility, and no osteoporotic treatment at discharge. Protective factors against mortality in the univariate analysis were surgical treatment by an experienced surgeon, management without an arthroplasty, allowing full weightbearing, mobilization in the first 48 hours postoperatively, and geriatric involvement. Risk factors for mortality in the multivariate analysis were cognitive impairment (Pfeiffer’s questionnaire) (hazard ratio (HR) 1.14 (95% CI 1.05 to 1.23), p = 0.002), age-adjusted CCI (HR 1.18 (95% CI 1.07 to 1.30), p = 0.001), and antiaggregant or anticoagulant medication at admission (HR 2.00 (95% CI 1.19 to 3.38), p = 0.009). Haemoglobin level at admission was protective against mortality (HR 0.85 (95% CI 0.74 to 0.97), p = 0.018).

**Conclusion:**

Mortality in FPIFs occurs mainly within the first six months of follow-up. Early co-management and clinical optimization, particularly targeting frail older patients, is crucial in reducing mortality following these fractures.

Cite this article: *Bone Jt Open* 2024;6(1):43–52.

## Introduction

Based on demographic trends and the increasing incidence of proximal femoral fractures, the prevalence of femoral peri-implant fractures (FPIFs) is expected to increase.^1-3^ This increase is linked to the growing population of individuals with non-prosthetic fixation devices, whose susceptibility to recurrent falls and new fractures grows with increasing age and comorbidities.^3,4^ We defined PIFs as a fracture occurring in bones with an existing non-prosthetic fixation device, such as plates, intramedullary nails, or screws.^4-9^

FPIFs, mainly involving older patients with multiple medical comorbidities,^10^ are associated with severe medical complications, prolonged hospital stays, and delayed recovery.^4,11^ The femoral periprosthetic fracture (FPPF) population shows mortality rates equal to or higher than those observed in the broader proximal femur fracture population,^1,10,12-19^ although FPIFs are distinct from FPPFs, and should be understood as a separate entity.

Challenges in the surgical treatment for FPIFs arise from factors related to the implant, the bone healing status, anatomical changes, and osteoporosis.^2,5,20,21^ Surgical treatment options and proposed algorithms are based on a relatively small number of patients.^5,6,21^ Most studies on FPIFs refer to cephalomedullary nails (CMNs), thus excluding diaphyseal and distal femoral implants.^1,3,4,6,10,20-23^ It is unknown whether different management methods affect mortality in FPIFs. Tools for decision-making are essential to reduce mortality and clinical complications, enhance functional outcomes, and preserve quality of life (QoL).

Given the rarity of FPIFs (0.5% to 2.3%),^1,3,6,7,11,22-25^ we conducted a multicentre, collaborative research project, Peri-Implant and Peri-Prosthetic fractures: AnalysiS (PIPPAS),^15^ to increase the number of patients included and provide more robust conclusions.^1,5,6,10,11^ This study aimed to evaluate the association between patient demographics, fracture characteristics, and fracture care on one-year mortality following FPIFs.

## Methods

The PIPPAS study is a collaborative, multicentre, prospective observational case series study (level IV evidence) evaluating PPFs and PIFs in 56 Spanish hospitals and one in Argentina.^15^ FPIF management was the standard of care at each participating site, as determined by the attending surgeon. We included patients aged 18 years or older who presented between January 2021 and November 2022 with a FPIF following nail or plate fixation, and available one-year follow-up clinical data. Fractures between an implant and a stemless femoral component of a knee prosthesis were included only if the fracture fixation device was a greater determinant for the surgical management than the knee prosthesis; otherwise, fractures between an implant and a stem were considered a FPPF. We excluded patients with pathological or intraoperative fractures, failed fixation without a new fracture line, such as cut-out or cut-in, and pregnancy. Written consent for participation in the study was obtained from all participants or their legal representatives.

**Fig. 1:**
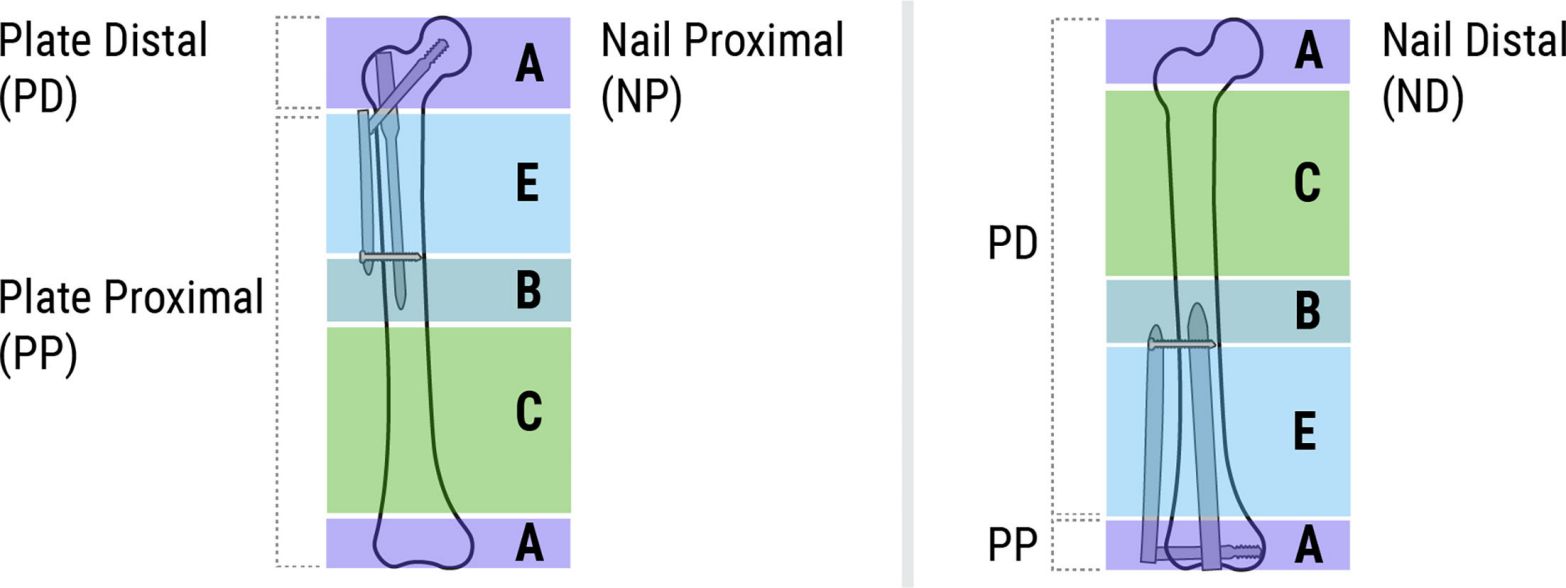
Diagram demonstrating the Broggi classification system for femoral peri-implant fractures, excluding type D fractures.

The index fracture refers to the fracture for which the implant related to the FPIF was originally used. Although several classifications have been used to describe FPIFs,^5,6,21,26^ many do not consider implants in the distal femur. We adopted the Broggi Classification for FPIFs,^26^ adding the healing status of the index fracture. We excluded Broggi-type D FPIFs from this analysis as they behave as a regular transverse subtrochanteric fracture, requiring screw removal (Figure 1).

Prospective data collection included patient demographics, management, and outcomes based on the Fragility Fracture Network’s Minimum Common Dataset for hip fracture audits but adapted to the specific nature of FPIFs (Supplementary Material).^27^ Cognitive status was assessed with the Pfeiffer Short Portable Mental Status Questionnaire (SPMSQ).^28^ Health-related QoL was assessed using the EuroQol five-dimension five-level questionnaire (EQ-5D-5L) instrument at six and 12 months.^29^ Experienced surgeons were those who had performed over 20 minimally invasive fixations or arthroplasty revisions in the last 12 months. Fracture healing was defined as the presence of at least three cortical callus bridges on radiological examination and pain-free full weightbearing. A comprehensive list of variables is available in the Supplementary Material.

Data were collected and managed using REDCap electronic data capture tools hosted at Instituto de Estudio de Ciencias de la Salud de Castilla y León in Spain.^30^ The manuscript was adapted to the STrengthening the Reporting of OBservational studies in Epidemiology (STROBE) statement. The study was conducted in accordance with the ethical standards laid down in the 1964 Declaration of Helsinki,^31^ and received approval from the institutional review boards of the coordinating centre and each participating hospital. This study is registered at ClinicalTrials.gov (NCT04663893).

The study involved 461 patients, of whom 440 met the inclusion criteria and 21 were lost to follow-up. The demographic and clinical characteristics of the participants are presented in Table I. Most patients were female (72.9%, n = 379), with a median age of 87 years (IQR 81 to 92). Most were frail, with a median Clinical Frailty Scale (CFS)^32^ of 6 (IQR 4 to 7), and mild cognitive impairment (median Pfeiffer SPMSQ 4 (IQR 1 to 7)). A large proportion were community-dwellers (72.5%, n = 317) and capable of walking outdoors (52.7%, n = 232).

**Table I.** Demographic and baseline data for patients presenting a femoral peri-implant fracture.

Variable	Value
**Total patients, n**	n = 440
**Median age, yrs (IQR)**	87 (81 to 92)
**Sex, n (%)**	
Female	363 (82.5)
Male	77 (17.5)
**Place of residence, n (%)**	
Own home	317 (72.5)
Nursing home	115 (26.3)
Hospital	5 (1.1)
N/A	3 (0.7)
Pre-fracture mobility,* n (%)	
1	63 (14.4)
2	73 (16.7)
3	96 (21.9)
4	111 (25.3)
5	95 (21.7)
N/A	2 (0.5)
**Pfeiffer’s SPMSQ**	
Median (IQR)	4 (1 to 7)
N/A, n (%)	27 (6.1)
**CFS**	
Median (IQR)	6 (4 to 7)
N/A, n (%)	10 (2.3)
**ASA grade, n (%)**	
I	6 (1.6)
II	81 (19.9)
III	250 (58.9)
IV	80 (19.2)
V	2 (0.5)
N/A	21 (4.8)
**Median CCI (IQR)**	6 (5 to 7)
**Osteoprotective treatment, n (%)**	
No treatment	250 (56.8)
Osteoprotective treatment	190 (43.2)
Anti-resorptive	64 (14.5)
Bone-forming	8 (1.8)
Calcium	121 (27.5)
Vitamin D	150 (34.1)
**Antiaggregant or anticoagulant medication, n (%)**	
None	289 (65.7)
Acenocumarol, NOAC, or PAA	145 (33)
Double	6 (1.4)
**Hb at admission**	
Median, gr/dL (IQR)	11.9 (10.6 to 13.1)
N/A, n (%)	3 (0.7)

* Pre-fracture mobility scale: 1 = complete independent gait; 2 = outdoors independent gait with one technical aid; 3 = outdoors independent gait with two technical aids; 4 = only indoors independent gait with or without aids; 5 = no mobility at all, or with the help of two other people.

ASA, American Society of Anesthesiologists; CCI, Charlson Comorbidity Index; CFS, clinical frailty scale; Hb, haemoglobin; N/A, not available; NOAC, new oral anticoagulant; PAA, platelet anti-aggregant; Pfeiffer’s SPMSQ, Pfeiffer’s Short Portable Mental Status Questionnaire.

### Statistical analysis

Quantitative variables were summarized as medians and IQRs, and qualitative variables were presented according to their frequency distribution. Multivariate Cox regression analyses were performed to identify independent risk factors for one-year mortality after FPIF, including variables that reached a significance level of 0.1 in previous univariate analyses. A chi-squared test was used to compare observed results with expected results. The hazard ratio (HR) indicates the increase of the mortality risk for each unit a quantitative variable increases. If the HR < 1 the variable is a protective factor. Kaplan-Meier estimators were used to estimate survival functions, and comparisons were made using the log-rank test. A p-value < 0.05 were considered statistically significant. Statistical analyses were performed using SPSS v.29 software (IBM, USA).

## Results

**Fig. 2:**
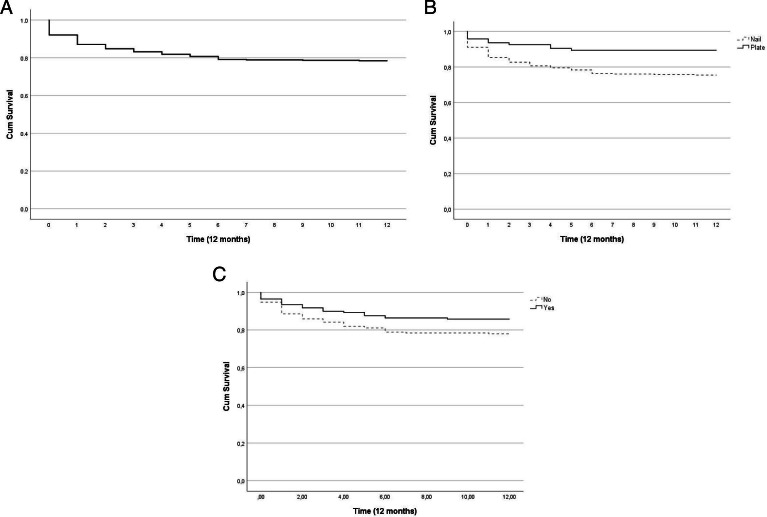
Kaplan-Meier curves for one-year mortality. a) Global mortality curve (95% CI 9.710 to 10.168). b) Mortality curves for femoral peri-implant fracture (FPIF) around a nail (95% CI 9.053 to 9.999) and FPIF around a plate (95% CI 10.251 to 11.558) mortality curves. c) Mortality curves for management strategies “implant removed” (95% CI 10.133 to 11.164) vs “implant retained” (95% CI 9.326 to 10.410).

Patient mortality rates were as follows: 8.2% (n = 36) in-hospital, 11.4% (n = 50) within 30 days, 21.1% (n = 93) within six months, and 21.6% (n = 95) within one year, as illustrated by Kaplan-Meier curves in Figure 2. The incidence rate of FPIFs during 2021 was 2.3/10^5^ individuals and 2.29% after femoral fracture fixation.

**Fig. 3:**
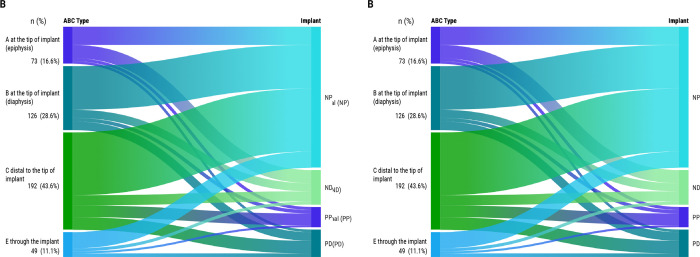
Sankey diagrams (made in Flourish; Canva, Australia) with femoral peri-implant fracture (FPIF) type distribution according to Broggi’s classification. a) Bone segment where the FPIF is located related to FPIF subtypes according to the implant. b) A, B, C, and E types related to FPIF subtypes according to the implant.

FPIF characteristics are detailed in Table II. The index fracture was already healed in 79.2% of cases. The type of FPIF according to Broggi’s classification is shown in Figure 3, with a greater risk of mortality for FPIFs around a nail than involving a plate (p = 0.007, univariate Cox regression analysis).

**Table II.** Femoral peri-implant fracture diagnostic features.

Variable	N (%)
**ABC type**	
A, at the tip of implant (epiphysis)	73 (16.6)
B, at the tip of implant (diaphysis)	127 (28.9)
C, distal to the tip of implant	193 (43.9)
E, through the implant	47 (10.7)
**Implant**	
Nail	345 (78.4)
Nail proximal	280 (63.6)
Nail distal	65 (14.8)
Plate	95 (21.6)
Plate proximal	39 (8.9)
Plate distal	56 (12.7)
**FPIF bone segment location**	
Proximal epiphysis	113 (25.7)
Diaphysis	227 (51.6)
Distal epiphysis	100 (22.7)
**Index fracture healed**	
No	90 (20.8)
Yes	343 (79.2)
**Previous infection**	
No	432 (98.2)
Yes	8 (1.8)
**Previous implant loosening radiological signs**	
No	423 (96.1)
Yes	17 (3.9)
**Implant displacement**	
No	395 (89.8)
Yes	45 (10.2)
**Time between index fracture to FPIF**	
< 1 mth	23 (5.4)
1 to < 6 mths	80 (18.8)
≥ 6 to < 12 mths	36 (8.5)
1 to < 5 yrs	114 (26.8)
≥ 5 yrs	172 (40.5)
N/A	15 (3.4)

FPIF, femoral peri-implant fracture; N/A, not available.

Management strategies are outlined in Table III. Most patients (90.5%, n = 398) were treated surgically, primarily under spinal anaesthesia (89.1%, n = 334) after a median delay of 85.8 hours (IQR 47.0 to 132.6). Less invasive surgical approaches were used in 48.7% (n = 194) of patients and primary implants were removed in 42.5% (n = 169) of the cases. Multiple fixation techniques were used, most frequently one single plate (45.7%, n = 182), and included overlapping techniques to prevent stress risers (57%, n = 227). For patients not managed with a prosthesis, the use of a cerclage for reduction, open approaches, or retaining the previous implant were associated with unrestricted postoperative weightbearing (all p < 0.001), with no influence on the one-year mortality (p = 0.327, p = 0.931, p = 0.054, respectively; chi-squared test).

**Table III.** Management of femoral peri-implant fractures. Categorical variables are summarized as absolute frequency and percentages from the number of patients managed surgically, except for “treatment”.

FPIF	N = 440
**Treatment, n (%)**	
Operative	398 (90.5)
Nonoperative	42 (9.5)
**Surgical delay, hrs**	
Median (IQR)	85.8 (47.0 to 132.6)
N/A, n (%)	2 (0.5)
**Type of anaesthesia, n (%)** *	
General	77 (17.5)
Spinal/regional	334 (89.1)
**Surgical approach, n (%)**	
Open	202 (50.8)
MIS	114 (28.6)
PC	80 (20.1)
N/A	2 (0.5)
**Removal of previous implant, n (%)**	
No	227 (57.0)
Yes	169 (42.5)
N/A	2 (0.5)
**Cerclage for reduction, n (%)**	
No	271 (68.1)
Yes	125 (31.4)
N/A	2 (0.5)
**Arthroplasty, n (%)**	
No	353 (88.7)
Yes*	43 (10.8)
N/A	2 (0.5)
**Type of fixation, n (%)** †	378 (95.0)
1 plate	182 (45.7)
2 plates	10 (2.5)
Nail	171 (43)
Ex fix	1 (0.3)
Cerclage	68 (17.1)
Isolated screws	9 (2.3)
**Overlapping, mm**	
Yes, n (%)	227 (57.0)
Median (IQR)	109 (61 to 154)
**Gap, mm**	
Yes, n (%)	17 (4.3)
Median (IQR)	25 (10 to 67)
**Kissing implants, n (%)**	4 (1)
**Interlocking, n (%)**	
No	315 (79.1)
Yes	74 (18.6)
N/A	9 (2.3)
**Surgeon experience, n (%)**	
> 20 arthroplasties	73 (16.6)
> 20 MIPO	153 (34.8)
**Medical staff involved in the patient’s care, n (%)** ‡	
No	75 (17)
Geriatrician	134 (30.5)
Internal Medicine	151 (34.3)
Geriatrician and others	51 (11.6)
Others	29 (6.6)
**Initial postoperative mobilization out of bed, n (%)**	
< 24 hrs	118 (27.8)
24 to 48 hrs	194 (45.8)
> 48 hrs	112 (26.4)
**Median total length of hospital stay, hrs (IQR)**	266.0 (184.1 to 386.1)

* Patients under spinal anaesthesia could also receive general anaesthesia. An arthroplasty was used as part of the FPIF treatment, but it was not recorded whether cement was used or not.

† The fixation strategy could include two or more fixation devices.

‡ Other than trauma or anaesthesia.

Ex fix, external fixator; FPIF, femoral peri-implant fracture; MIPO, minimally invasive plating ostheosynthesis; MIS, minimally invasive surgery; N/A, not available; PC, percutaneous.

Complications and secondary outcomes other than mortality are outlined in Table IV. Most fractures healed, with a nonunion rate of 4.1% (n = 18) at one year; only five patients (1.1%) were surgically treated for nonunion. An improvement in QoL was noted between six- and 12-month follow-up. Medical complications were commonly present in the acute episode, particularly delirium. After hospital discharge, pulmonary complications were the most common. Surgical complications mainly involved dislocations and prosthetic loosening among patients treated with arthroplasties, while patients managed with fixation had complications related to fracture healing and fixation failure. However, there were no differences in overall complications between both groups.

**Table IV.** Postoperative care and follow-up data for femoral peri-implant fractures. Categorical variables are summarized as absolute frequency and percentages from the number of patients in each category.

Medical complications	In-hospital	30 days	6 mths	12 mths
No	194 (47.4)	326 (81.5)	233 (71.5)	209 (80.4)
Yes (any)	215 (52.6)	74 (18.5)	93 (28.5)	51 (19.6)
Cardiac	59 (27.4)	13 (3.3)	20 (6.1)	13 (5)
Pulmonary	52 (24.2)	20 (5)	27 (8.3)	23 (8.8)
Pulmonary thromboembolism	3 (1.4)	0 (0)	1 (0.3)	0 (0)
Renal	65 (30.2)	9 (2.3)	8 (2.5)	5 (1.9)
Cerebral	6 (2.8)	8 (2)	7 (2.2)	2 (0.8)
Gastrointestinal	43 (20)	12 (3)	12 (3.7)	7 (2.7)
Urinary tract infection	47 (21.9)	13 (3.3)	10 (3.1)	7 (2.7)
Delirium	97 (45.1)			
In-hospital fractures	5 (2.3)			
Other medical complications		10 (2.5)	25 (7.7)	21 (8.1)
**Surgical complications**				
No		377 (94.3)	296 (90.8)	244 (93.8)
Fracture in the same bone		2 (0.5)	7 (2.1)	4 (1.5)
Failure of fixation		1 (0.3)	1 (0.3)	1 (0.4)
Dislocation (prosthesis)		16 (4)	14 (4.3)	5 (1.9)
Loosen prosthesis		7 (1.8)	7 (2.1)	2 (0.8)
Infection		0 (0)	0 (0)	0 (0)
Nonunion				5 (1.9)
**Weightbearing restrictions**	**Hospital discharge**			
No restrictions	165 (40.3)	176 (45.1)		
Only for transferences	55 (13.4)	75 (19.2)		
Not allowed	176 (43)	137 (35.1)		
N/A	13 (3.2)	2 (0.5)		
Osteoprotective treatment*				
No treatment	157 (38.4)	167 (43.0)	123 (42.3)	97 (40.4)
**Osteoprotective treatment**	252 (61.6)	221 (57.0)	168 (57.7)	143 (59.6)
Antiresorptive	94 (37.3)	93 (42.1)	68 (40.5)	51 (35.7)
Bone-forming	24 (9.5)	20 (9.0)	15 (8.9)	16 (11.2)
Calcium	153 (60.7)	147 (66.6)	110 (65.5)	92 (63.3)
Vitamin D	180 (71.4)	186 (84.2)	142 (84.5)	118 (82.5)
**Place of residence**				
Home	210 (51.3)	218 (56.0)	194 (66.7)	166 (69.5)
Nursing home	151 (36.9)	150 (38.6)	91 (31.6)	71 (29.7)
Hospital	12 (2.9)	21 (5.4)	5 (1.7)	2 (0.8)
**EQ-5D**			0.608 (0.575 to 0.641)	0.694 (0.661 to 0.727)

* The percentages for the different osteoprotective treatments refer to the total number of patients who were receiving treatment.

EQ-5D, EuroQol five-dimension questionnaire.

Univariate analysis identified that patients who died were older, more frail (higher CFS, ASA score, and CCI), non-community dwellers, dependent ambulators, and did not receive osteoporosis treatment. Fractures in the distal epiphysis, around a proximal nail (vs a distal nail or a distal plate), first mobilization more than 48 hours after the operation, weightbearing restrictions, not involving a geriatrician, discharge to a skilled care facility, and not treating osteoporosis at discharge were associated with mortality; while FPIFs around a plate, surgical treatment, or surgery done by an experienced surgeon were protective factors (Supplementary Table i). Multivariate analysis showed that cognitive impairment, CCI, and preoperative antiaggregant or anticoagulant treatment were risk factors for mortality during the first-year post-fracture, whereas the haemoglobin level at admission was protective (Table V).

**Table V.** Cox’s regression multivariate analysis.

Variable	p-value	HR	95% CI for HR
Removal of previous implant: yes vs no	0.228	0.723	0.427 to 1.225
Cognitive impairment (Pfeiffer’s SPMSQ)	0.002	1.135	1.048 to 1.229
CCI (age-adjusted)	0.001	1.178	1.067 to 1.301
Haemoglobin at admission, g/dl	0.018	0.850	0.743 to 0.973
Antiaggregant or anticoagulant medication at admission (no ref)	0.033		
Either Acenocumarol or NOAC or PAA	0.009	2.000	1.185 to 3.376
Double	0.414	1.861	0.419 to 8.253

CCI, Charlson Comorbidity Index; HR, hazard ratio; NOAC, new oral anticoagulant; PAA, platelet antiaggregant; Pfeiffer’s SPMSQ, Pfeiffer’s Short Portable Mental Status Questionnaire.

## Discussion

To the best of our knowledge, this is the largest study on FPIFs assessing risk factors for one-year mortality. Despite their rarity, FPIFs are a severe complication in this elderly and frail population.^3^ The working hypothesis in most studies on FPIFs is whether short and long CMN have similar FPIF rates, resulting in a small reported number of FPIFs. Previous studies have reported incidences between 1.4% and 2.0%,^1,24^ with a slightly higher rate of 2.3% observed in Spain, likely due to the country’s higher life expectancy. The rate of FPIFs around CMN is decreasing with newer generations of nails.^25^

Compared to other studies on FPIFs, patients in this study were older, with a greater proportion of females,^1,3-6,21,24,33^ likely attributable to higher life expectancy in Spain. Information on patients’ comorbidities or fracture baseline data is scarce; in the study by Jennison and Yarlagadda^10^ patients were aged 12 years younger, but with worse ASA grades. Frailty, limited mobility (no outdoor ambulation), and living in nursing homes were candidate predictors of mortality in the univariate analysis. Like the PIPPAS study, previous studies report that 70% to 80% of index fractures had already healed at the time the FPIF occurred.^1,5,7,11,22^ Only Bidolegui et al^21^ and Lindvall et al^23^ presented all index fractures healed. Typically, FPIFs occurred more than one year after the index fracture fixation,^3,4,6,7,22,34^ except in rare cases where they happened after 1.5 months.^24,25^

The nail-to-plate ratio in this study was 4:1, but this ratio varies across literature from 1:1 to 1:2.5,^5,6,21^ influenced by the treatment chosen for the index fracture. Some authors only report FPIFs on nails and exclude plates.^7,22,33^ As in most series, the most involved segment was the diaphysis, with fractures located at the tip of the implant.^1,5,6,21,23,33^ Distal FPIFs, particularly type C fractures occurring distal to a proximal nail or in the distal metaphysis, were associated with increased mortality. As in this study, Müller et al^1^ found that mortality was higher for nail-related FPIFs.

One-year mortality rates vary across studies, ranging from 18.6% to 44.8%.^1,3,7,10,11^ As in Lang et al,^11^ patients mainly died within the first six months. Jennison and Yarlagadda^10^ reported a double mortality rate, with 23% of 29 patients deceased at one-month follow-up. These differences can be explained by the heterogeneity of the studies. FPIF mortality is higher than that in hip fracture patients; the latter has benefited from practices to improve outcomes including national hip fracture audits.^10^ PIPPAS aims to raise awareness that patients with FPIFs would also benefit from these measures.

Some authors failed to find significant risk factors for mortality in patients with FPIF.^10,11^ Prompt surgical treatment, and lower CCI^10^ and ASA grades,^11^ have shown a trend towards survival. This study confirmed that reduced general health status, as evidenced by higher CCI, cognitive impairment or antiaggregant/anticoagulant medication, and lower haemoglobin levels at admission, are risk factors for mortality. Optimization of health status upon arrival, including anaemia, can help reduce mortality. Surgical management is usually discarded in patients deemed unfit for surgery. Clinical optimization and prompt management of medical complications can be better achieved with geriatric co-management.

Strategies that are protective candidate predictors should be considered during fracture care: surgical treatment by an experienced surgeon, removing the implant, with a new fixation allowing full weightbearing and early postoperative mobilization while co-managed with a geriatrician. The reason for removing the previous implant was mostly to use intramedullary devices, i.e. a nail or a prosthesis. Clinically, it is unclear whether or not removing the implant is better for patient survival, especially if different appropriate treatment alternatives exist that do not require implant removal. For example, current treatment options for diaphyseal FPIFs distal to CMN could be either adding a plate or changing the nail for a longer nail. Goodnough et al^33^ found that revision to a longer nail for FPIFs around a CMN in 16 patients was associated with increased patient morbidity. Our study, by contrast, found a trend towards removing the implant as a protective candidate predictor against mortality, but included FPIFs around plates and nails. It is important to consider that if the index fracture remains unhealed, and the patient then suffers a second fracture (the FPIF), then there will be two fracture sites in the same bone. Both fracture sites need adequate fixation in order to heal, although the healing status of the index fracture had no influence on mortality.

Patients who were not mobilized promptly after surgery, or without unrestricted weightbearing, were also more prone to be discharged to skilled care facilities. Medical or surgical complications at any time were risk factors for mortality. Post-fracture osteoporosis treatment protected against mortality in the univariate analysis; such treatment could be a confounding factor, as poly-medicated patients with more comorbidities are less likely to start treatment for osteoporosis. Furthermore, patients treated by geriatricians, who are more prone to initiate osteoporosis treatment, were more likely to survive.

Several classification systems for PIFs have been proposed, but none have gained widespread acceptance.^3-6^ The original FPIF classification by Videla-Cés et al^26^ has been validated, with a high inter- and intraobserver correlation rate. Treatment algorithms based on FPIF classifications are unable to compare outcomes of different operative strategies^4,5^ and encourage future multicentre research to study expected outcomes.^6^ The PISCO group presented a consensus review on FPIF treatment with expert recommendations, but it is yet to be determined whether they reduce mortality and complication rates.^9^

The aims of treatment are ‘getting it right first time’ with a single operation, which allows immediate unrestricted weightbearing with a low risk of complications, and avoids the creation of stress risers locally which may predispose to further PIFs, using a long implant to span the entire femur, or at least overlap the original implant.^2,5^ There is a wide range of surgical strategies,^4,7,11,24^ with little information on mortality and functional outcomes regarding walking ability and place of residence. Lang et al^11^ recommended the use of cerclage wires as a reduction tool through an open approach. However, to avoid potential harm, we recommend less invasive approaches. Poroh et al^4^ reported a nonunion rate of 4.2%, similar to the 4.1% observed in the present study, while in other studies all FPIFs healed.^1,5,21,33^

The present study found an association of demographics, fracture characteristics, and treatment strategy with one-year mortality following FPIFs. However, a detailed analysis of the treatment options for each fracture type, including implant and fracture location, could provide more well-defined recommendations on surgical management.

Limitations of this study include, first, its heterogeneous nature in terms of implants used, FPIF locations, and healing status of the index fractures, which complicates the formulation of conclusive general guidelines. Second, there were no monitoring visits of the participating sites, and no external confirmation to limit the number of unconscious errors each site may have made when entering data in REDCap. Lastly, a more detailed analysis of specific FPIF patterns could give more precise guidance for surgical treatment strategy. Despite these limitations, a comprehensive understanding of these fractures can assist readers in organizing their resources.

In conclusion, mortality associated with FPIF predominantly occurred within the first six months of follow-up. Risk factors for mortality were cognitive impairment, age-adjusted CCI, and antiaggregant/anticoagulant medication at admission. Conversely, higher admission haemoglobin levels proved to be protective against mortality. Implementing early geriatric co-management and clinical optimization strategies, especially for frail patients, can enhance survival outcomes in FPIF cases.

**Take home message**

- Mortality rate associated with femoral peri-implant fractures (FPIFs) was 21% and predominantly occurred within the first six months of follow-up.

- Risk factors for mortality in FPIFs were cognitive impairment, age-adjusted Charlson Comorbidity Index, and antiaggregant/anticoagulant medication at admission.

- Early geriatric co-management and clinical optimization strategies can enhance survival outcomes in FPIF patients.

## Data Availability

The datasets used and/or analyzed during the current study are available from the corresponding author on reasonable request.
